# Errata

**Published:** 2012-06-18

**Errata 1**

CERTIFICATION

Hereby certifies that the articles Highlights in the minimally invasive treatment of SUI in women, authors Surcel C, Chibelean C, Iordache A, Mirvald C, Gngu C, Margaritis S, Stoica R, Codoiu C, Savu C, Marksteiner R, Sinescu I, published in vol 4, issue 3, Journal of Medicine and Life and Minimally invasive treatment for female stress urinary incontinence Romanian highlights authors Surcel C, Chibelean C, Iordache A, Mirvald C, Gngu C, Margaritis S, Stoica R, Codoiu C, Savu C, Marksteiner R, Sinescu I, published in vol 4, issue 4, Journal of Medicine and Life, appeared at one issue distance, have been published as a result of lack of communication between the authors of the articles and the reviewers of Journal of Medicine and Life, and, as a consequence of the previous Publishing Editors leaving in UK for doctoral studies, the Publishing Editor who took her place and whose task was to review the article, was not familiarized with the publishing and editing procedures specific to Journal of Medicine and Life. 

Prof. I. Sinescu, MD, had no implication in the relationship with Carol Davila Printing Press and in the process of publishing the above-mentioned articles. 

Therefore, Carol Davila University Press takes the blame for these dysfunctions and considers appropriate the withdrawal of the two above-mentioned articles from PubMed and the archive of the journal (website and printed versions). 

The University Press who is the owner of Journal of Medicine and Life regrets the possible prejudices suffered by the authors by the publishing the two articles (revised versions of a single article) and wishes to continue the collaboration with the authors. 

**Errata 2**

The following article has been published both in English in Journal of Medicine and Life and in Romanian in Revista Romana de Urologie. The article Management of small renal masses update 2011, authors Surcel C, Mirvald C, Gngu C, Stoica R, Sinescu I, published in Journal of Medicine and Life, 2011 May 15, 4(2): 139-47, ISSN: 1844-122x (CNCSIS B+ 852) is similar to Managementul tumorilor renale parenchimatoase mici, authors Sinescu I, Surcel C, Cngu C, Mirvald C, Stoica R, Tuca M, published in Revista Romana de Urologie, 2010, vol. 9, nr. 4, ISSN: 1223 0650 (CNCSIS B+ 486).

The English version of the article published in Journal of Medicine and Life should be removed (deleted) from the printed/ website versions of Journal of Medicine and Life and PubMed database.

**Errata 3**

The following article has been published both in English in Journal of Medicine and Life and in Romanian in Revista Romana de Urologie. The article Prognostic factors in retroperitoneal fibrosis, authors I. Sinescu, C. Surcel, Mirvald C, Chibelean C, Gngu C, Avram D, Hrza M, Manu MA, Lazar R, Savu C, Udrea A, published in Journal of Medicine and Life, 2010, January-March, 3(1), 19-25, ISSN: 1844-122x (CNCSIS C 852), PMID: 20302193 (PubMed indexed for MEDLINE) is similar to Factori de prognostic in fibroza retroperitoneala idiopatica, authors Sinescu I, Surcel C, Mirvlad C, Chibelean C, Cngu C, Avram C, Hrza M, Manu MA, Lazar R, Savu Carmen, Gutu I, Udrea A, Joianu S, published in Revista Romana de Urologie, 2009, vol. 8, nr. 4, 53-59, ISSN: 1223-0650 (CNCSIS B+ 486).

The English version of the article published in Journal of Medicine and Life should be removed (deleted) from the printed/ website versions of Journal of Medicine and Life and PubMed database.

**Errata 4**

In the article Complete spontaneous regression of a total pneumothorax in a patient with chronic obstructive lung disease by Gonlugur U, Mirici A, Yildiz M, which appeared in Volume 4, Issue 4, October-December 2011, of Journal of Medicine and Life (J Med Life. 2011 Nov 14;4(4):419-20. Epub 2011 Nov 24), the names and given names of the authors were changed. 

The authors names should have appeared as it follows: Gonlugur U, Mirici A, Yildiz M.

**Errata 5**

In the article Primary tubercular abscess of the breast an unusual entity by Gupta R, Singal RP, Gupta A, Singal S, Shahi S, Singal R, which appeared in Volume 5, Issue 1, January-March 2012, of Journal of Medicine and Life (J Med Life. 2012 Feb 22;5(1):98-100. Epub 2012 Mar 5. [PMID: 22574095]), the name of the second author was misspelled.

The complete list of authors should have appeared as it follows: Gupta R, Singal RP, Gupta A, Singal S, Shahi S, Singal R.

**The publishers regret any inconvenience caused by these errors.**

